# Exploring the implications of distinct mutational signatures and mutation rates in aging and cancer

**DOI:** 10.1186/s13073-016-0286-z

**Published:** 2016-03-17

**Authors:** Edward J. Fox, Jesse J. Salk, Lawrence A. Loeb

**Affiliations:** Department of Pathology, University of Washington, Seattle, WA 98195 USA; Department of Medicine, Division of Oncology, University of Washington, Seattle, WA 98195 USA; Department of Biochemistry, University of Washington, Seattle, WA 98195 USA

## Abstract

Signatures of mutagenesis provide a powerful tool for dissecting the role of somatic mutations in both normal and pathological processes. Significantly, cancer genomes are dominated by mutation signatures distinct from those that accumulate in normal tissues with age, with potentially important translational implications.

## Deconvoluting mutational signatures in the cancer genome

The initiation and progression of human cancers are fueled by mutations and driven by adaptive selection. Consequently, many of the heritable phenotypic changes required to transform normal cells become etched in the cancer genome. Advances in high-throughput sequencing over the past decade have brought a greater appreciation for the number and types of DNA alterations that accumulate during cancer evolution. These somatic alterations are typically classified as either “driver” mutations, which confer selected cancer phenotypes, or the far more numerous “passenger” mutations, which hitch-hike with driver mutations but in and of themselves are not thought to be directly selected. Both the multiplicity and heterogeneity of these changes have unfortunately confounded attempts to reduce the evolutionary complexity of most cancers to a targetable number of recurring driver mutations. Additionally, whether normal mutation rates and processes are sufficient to explain the mutation patterns found in cancer has been debated for decades. In the last few years, DNA sequence analyses of cancer genomes have allowed the detection of distinct mutational signatures and, more recently, those occurring during aging. These findings have implications for our understanding of cancer initiation, evolution, and potentially for therapy.

Stratton and colleagues at the Sanger Center, among others, have recently developed bioinformatic and computational tools to deconvolute mutational signatures that are over-represented in cancer genomes [1, 2]. Building on a large biochemical literature, they have inferred that some of the distinct mutational processes operative during the evolution of a tumor can be resolved by analyses of the bases immediately 5′ and 3′ of the mutated base [3]. Their initial studies show that more than 20 mutational signatures, representing a diversity of mutational processes underlying the development of cancer, can be identified across multiple cancer types.

Unlike cancer cells, normal cells replicate their genomes with extraordinary accuracy and have an armamentarium of postreplicative DNA repair pathways to ensure genetic stability. Neither DNA replication nor postsynthetic DNA repair is, however, without error and some of the more than 50,000 estimated DNA damage events which occur daily within each normal cell can result in mutation. The majority of these mutations, destined to become passenger mutations should the resulting lineage ever be expanded, confer no demonstrable phenotype. The catalog of mutations present in a cancer genome at the time of sampling, therefore, is the sum of “normal” mutational processes occurring from the time an egg is fertilized and mutational processes occurring in tumors from the time a cancer is initiated. Some of these normal mutagenic processes will occur intermittently, e.g., caused by exposure to environmental mutagens, while others, such as replication errors, would be predicted to generate mutations at a more constant rate.

Now, using data from The International Cancer Genome Consortium (ICGC) of 10,250 cancer genomes across 36 cancer types, Alexandrov et al. have extended these analyses to deconvolute from cancer genomes the signatures of mutations that likely occurred during normal aging from those that were acquired during malignant transformation [4]. They identified two mutational signatures, designated Signatures 1 and 5, for which the number of attributable mutations was proportional to the chronological age of the cancer patient at diagnosis. Interestingly, both signatures exhibit different mutation frequencies in different tissues and are not themselves correlated; only three cancer types show an age-correlation for both signatures (i.e., breast cancer, low-grade glioma, and medulloblastoma). This is likely to suggest that the underlying mechanisms of mutation are different for each signature.

Signature 1 is largely made up of C>T substitutions at CpG dinucleotides, which are likely caused by spontaneous deamination of 5-methylcytosine, enzymatic deamination of cytosine, or polymerase errors [5]. The cancer types exhibiting high Signature 1 mutation frequencies are chiefly derived from epithelia with high turnover and the signature may well mark the number of cell replications. Signature 5 primarily features a combination of C>T and T>C transitions, exhibits a degree of transcriptional strand bias, and may result from an endogenously generated metabolite(s). In contrast to Signature 1, the frequency of Signature 5, which also displays substantial variation between cancer types, is highest mainly in cancers of the brain, kidney, and thyroid.

## Mutation rates and cancer initiation

A striking finding of the studies is that no more than a quarter of the mutations found in cancer genomes exhibit any correlation with age or carry signatures of the physiological mutagenic processes operative during normal aging. Whether the mutation rate of cancer cells is elevated relative to normal has been continuously debated since the original description of the mutator phenotype hypothesis [6]. Defects in pathways governing genetic stability were reasoned to facilitate tumorigenesis by fueling the reiterative process of mutation, selection, and clonal expansion that drives cancer progression. One of the earliest and most emphatic conclusions of cancer genome sequencing studies was that, as predicted by the mutator phenotype hypothesis, cancers carry vast and varied numbers of mutations.

The quantification of the relative contribution of physiological mutagenic processes to the cancer genome now reinforces the concept that elevated rates of mutation accumulation underpin the cancer phenotype. The distinct mutational signatures of age-associated and tumor-associated mutations also emphasize the fundamental differences between the mechanisms shaping normal and cancer genomes. Importantly these findings are also inconsistent with the proposal that the majority of risk among different tissues to the development of tumors can be explained by the number of normal stem cell divisions in those tissues [7]. The large numbers of mutations found in cancers was originally cited in support of this; however, the lack of a common and dominant age-related mutational signature argues against random mutations arising during DNA replication in normal, non-cancerous stem cells as the chief source of mutagenesis. The differences in age-related mutational signatures between different tissues must represent intrinsic differences in the lifetime exposures to endogenously and exogenously generated DNA damaging agents as well as differences in the postsynthetic DNA repair and replicative capacities of different cell types.

## Implications for medicine

Aging is the greatest risk factor for malignancy. Modulating the mutagenic processes underlying Signatures 1 and 5 could therefore offer the possibility of delaying cancer initiation and other age-related pathologies. Additionally, changes in mutation signatures might aid in early cancer diagnosis or in the quantification of genotoxic exposures especially through the application of “liquid” biopsies. The results of Alexandrov et al. suggest that, in contrast to the prevailing focus of oncology on blocking key signaling hubs in growth pathways of tumor cells, mutation accumulation itself represents an underappreciated therapeutic target. As high levels of mutation are potentially deleterious, it has been proposed that the mutation frequency of the cancer genome is thresholded at a level below which cancers can maximize their genetic diversity but still maintain fitness, i.e., the population avoids undergoing an “error catastrophe” [8]. In addition, while it is known that prognosis and mutation load are correlated in both colorectal and endometrial cancers, it is increasingly being appreciated that a high neoantigen load, secondary to a high mutation load, is also a major determinant of response to immunomodulatory therapies in certain cancers [9]. These observations have led to the proposal that both the frequency and rate of mutation of cancer cells, rather than individual driver mutations, might be an effective target. Treatment of cancer cells with mutagenic nucleoside analogs, for example, might result in ablation of the tumor through induction of an immunologically mediated error catastrophe [8].

Thus far, the identification of specific mutational signatures attributable to specific biological processes is largely correlative; of particular note, the age-correlated signatures identified by Alexandrov et al. were obtained from tumor genomes and not from histologically normal, aged tissues. Further analyses and experimentation in model organisms are required before one can infer a causal mechanism. It will be necessary, for example, to modulate the rate-limiting step in specific biological pathways to determine if this results in alterations of the corresponding mutational signature. Ultimately, the direct investigation of in vivo somatic mutation rates and signatures, for both tumor and normal tissue, will require accurate sequencing of single cells and molecules [10].

Cancer is first and foremost a disease of DNA, where evolutionary changes are driven by selection of stochastically generated pre-existing variants. It has long been argued that the rates and mechanisms of mutation operative in normal cells cannot account for the large numbers and types of mutations found in cancer and that defects in pathways governing genetic stability must be dysregulated to facilitate tumorigenesis. For the first time, it has become possible to quantify mutational signatures that likely occurred during normal aging relative to those that were acquired during malignant transformation and tumor progression. The capacity to accumulate mutations, rather than to mutate any particular subset of genes, has once again been confirmed as one of cancer’s key hallmarks.
